# Climate change and human health in the urban environment of the Eastern Mediterranean and Middle East: A workshop report

**DOI:** 10.3934/publichealth.2026047

**Published:** 2026-08-24

**Authors:** Fragkeskos Kekkou, Niki Paisi, Aayushi A. Sharma, Kamil Erguler, George K. Christophides, Jos Lelieveld, Marco Neira

**Affiliations:** 1 Climate and Atmosphere Research Center (CARE-C), The Cyprus Institute, Nicosia, Cyprus; 2 Department of Life Sciences, Imperial College London, London SW7 2AZ, UK; 3 The Cyprus Institute, 20 Konstantinou Kavafi Street, Nicosia 2121, Cyprus; 4 Max Planck Institute for Chemistry, Mainz, Germany

**Keywords:** climate change, human health, Middle East, Eastern Mediterranean, heat, extreme temperature, air pollution, air quality, disease vector, vector-borne diseases

## Abstract

The Eastern Mediterranean and Middle East (EMME) is a region comprised of 18 countries that are home to approximately 460 million people. This region spans across three continents and has been identified as a hotspot for climate change. As part of ongoing efforts to understand the potential impacts of climate change on public health in this part of the world, the scientific workshop “Climate and Health in the Urban Environment” was held on September 24–25, 2024, in the cities of Nicosia and Larnaca (Cyprus). The workshop brought together regional experts from diverse fields and aimed to assess emerging health risks, identify research gaps, and propose policy interventions with a focus on urban environments in the EMME region. Specific thematic sessions covered the topics of extreme temperatures, vector-borne diseases, and air quality. Here, we present a synthesis of the talks, discussions, and outcomes from the workshop, highlighting the latest findings on climate-related health risks in the EMME. Furthermore, we propose a series of recommendations for informing research and policy decisions in the region.

## 1. Introduction

The Eastern Mediterranean and Middle East (EMME) is a region that spans three continents —Europe, Asia, and Africa— contains 18 countries, and is home to approximately 460 million people [1]. The countries that make up the EMME are shown in Figure 1 (basemap source: [2]). The population structure and socioeconomic conditions within the region are highly heterogeneous, with marked disparities in income and infrastructure, translating into varied capacities to adapt to environmental stresses. Recognized as a global climate change hotspot, the EMME faces three critical challenges with substantial implications for public health: extreme heat, vector-borne diseases, and air pollution.

**Figure 1.**
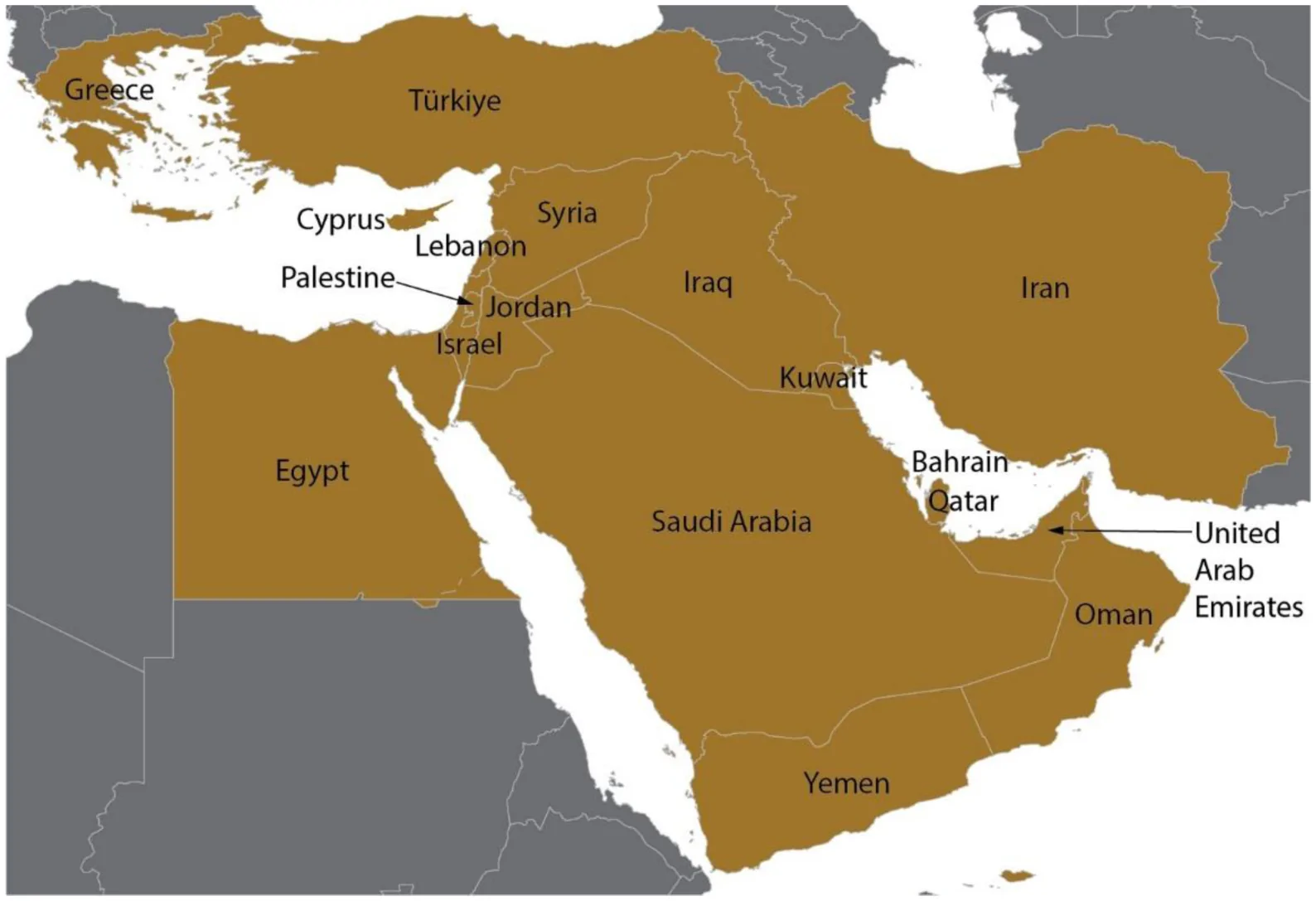
The EMME region. Countries included in the EMME are colored brown and labeled. Non-EMME countries are colored in gray.

Over recent decades, average temperatures in the EMME have risen nearly twice as fast as the global average, accompanied by a notable increase in the frequency and severity of extreme weather events, including heatwaves, prolonged droughts, dust storms, and flash floods. Under high-emission scenarios, regional temperatures are projected to rise approximately 3–5 °C by mid-century, and potentially exceed 5 °C by 2100, significantly surpassing global average warming projections [3].

In parallel with rapid demographic growth, urbanization rates have surged in this region, with data from the United Nations revealing that approximately 65%–72% of the EMME population was residing in urban areas by the late 2010s (well above the global average, ~55%), and projections indicating that nearly all countries within the region will become predominantly urbanized by the mid-21st century [4]. This rapid urban expansion, combined with already strained infrastructure and social systems, exacerbates the vulnerability of local populations [5]–[7], particularly impoverished and displaced communities [8],[9].

Several urban centers across the region have begun experiencing record-breaking temperatures, and future projections suggest that rising temperatures and humidity may increasingly challenge human survival in the coming decades [10]–[14]. Vulnerable groups, including the elderly, children and adolescents, individuals with preexisting medical conditions, and outdoor workers, are at increased risk for heat-related health issues such as heat stroke, renal failure, and cardiovascular diseases [15]–[18], as well as adverse mental and behavioral health outcomes [19] (Figure 2).

**Figure 2.**
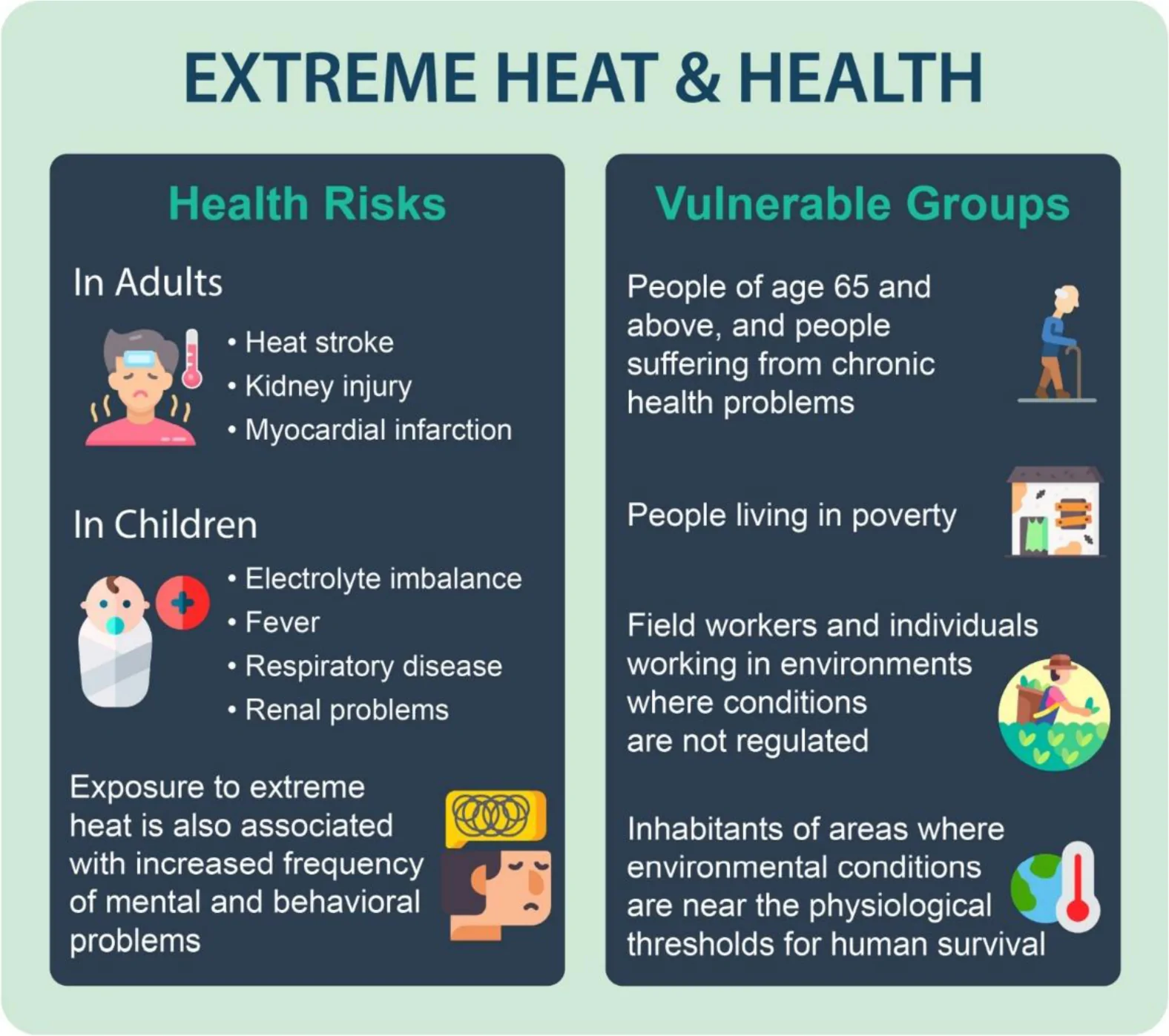
Health risks and vulnerable populations associated with extreme heat exposure. Acute outcomes include heat stroke, kidney injury, and myocardial infarction in adults, as well as electrolyte imbalance, fever, respiratory disease, and renal dysfunction in children. Populations at heightened risk comprise the elderly, the chronically ill, those living in poverty, outdoor workers, and residents of areas approaching human physiological heat-stress limits.

Deteriorating air quality, driven primarily by fossil fuel combustion, urban growth, and frequent dust storms, further complicates the public health landscape [20]–[22]. Elevated levels of pollutants like PM_2.5_, PM_10_, and ground-level ozone have been linked to respiratory conditions, cardiovascular diseases, and cancers [23]. Globally, air pollution is estimated to cause approximately eight million excess deaths annually, over one million of which occur within Europe and the Eastern Mediterranean region [22],[24].

Shifting climatic conditions in the EMME, such as altered temperature and precipitation patterns, facilitate the proliferation of vector species like *Aedes aegypti* and *Aedes albopictus*, responsible for the transmission of vector-borne diseases (VBDs) like dengue fever, Zika, and chikungunya [25]–[28]. The epidemiological landscapes of parasitic VBDs, such as malaria and leishmaniasis, are also expected to change, potentially leading to their emergence or re-emergence in various parts of the region [25],[29].

As part of ongoing efforts to address these climate-related public health challenges, the scientific workshop “Climate and Health in the Urban Environment” was convened in a hybrid format on September 24–25, 2024, in the cities of Nicosia and Larnaca, Republic of Cyprus. Conducted within the framework of the Conference “The Climate Crisis in the Eastern Mediterranean and the Middle East” (https://emmeclimate2024.cyi.ac.cy/), the workshop facilitated interdisciplinary dialogue among experts from diverse fields. Specifically, the workshop included a total of 19 invited speakers, with 15 attending in person and four participating online. Participants were invited based on their expertise and active research contributions within the three thematic pillars of the event (extreme heat and human health, air quality and human health, and vector-borne diseases).

The workshop aimed to identify research gaps, assess emerging health risks, and outline policy interventions specifically targeting urban environments in the EMME. Through interdisciplinary dialogue, the workshop provided a platform for knowledge exchange and collaborative action to strengthen climate resilience and public health strategies. To this end, experts were selected to provide complementary coverage across the three aforementioned thematic pillars and were explicitly asked to address research gaps and present policy suggestions relevant to their respective fields of expertise during their interventions. Each thematic session concluded with a dedicated discussion period in which all attending experts were invited to debate the priorities raised by the session's speakers and provide input from their own disciplinary and regional perspectives, with designated rapporteurs documenting the outcomes. As a result, the gaps and recommendations synthesized in this report reflect a collective output of the workshop, rather than the views of individual presenters in isolation.

Although the event was conceived as an EMME-wide convening, logistical constraints (e.g., travel restrictions and funding asymmetries), as well as the uneven distribution of research institutions and infrastructure across the region, made it challenging to achieve an even geographic representation of researchers from the entire region. While attending speakers were affiliated with institutions from Cyprus, Greece, Israel, the United Kingdom, Germany, Spain, and Tunisia (and their research projects often extend to other countries in the EMME), regions such as the Arab Gulf states, Iran, Egypt, Yemen, and the broader Levant were unfortunately not directly represented in the workshop. In drafting this manuscript, we have sought to mitigate this imbalance to some degree by drawing on published scientific literature focused on the underrepresented countries. We further address this limitation in the Concluding Remarks section.

## 2. Extreme temperatures

The workshop's first session focused on the effects of both extreme daytime and nighttime temperatures, the influence of weather patterns on seasonal health outcomes, and how advancements in heat-health modeling and early warning systems (EWS) can help mitigate these concerns. Additionally, presenters discussed the challenges associated with data availability, modeling uncertainties, and regional differences in adaptability.

Presentations were offered by Marco Neira (The Cyprus Institute, Cyprus), Robin Fears (Advisor to the InterAcademy Partnership), Christos Giannaros (National and Kapodistrian University of Athens, Greece), Itamar Grotto (Ben-Gurion University of the Negev, Israel), Hila Oren (Tel-Aviv University, Israel), Anastasia Paschalidou (Democritus University of Thrace, Greece), Dominic Royé (Climate Research Foundation, Madrid), and Assaf Hochman (Institute of Earth Sciences, Hebrew University of Jerusalem, Israel).

### 2.1. Escalating heat risks in the EMME region

The EMME is warming at a faster rate than the global average, leading to exacerbated health risks for vulnerable populations [14]. During the workshop, Robin Fears highlighted new evidence underscoring the growing challenges in the EMME region. Referring to the 2024 UN report, he expressed concerns about rising heat-related mortality due to recent record-breaking temperatures and the more frequent and intense heatwaves in the Middle East, resulting in hotter and drier conditions [30]. Furthermore, he addressed the increasing intensity of the urban heat island (UHI) effect in the Eastern Mediterranean and Middle East regions, amplifying health risks in densely populated cities [31]–[33]. While some populations have managed to acclimatize to high temperatures, future projections show that heat stress will exceed physiological and infrastructural adaptation limits, particularly for vulnerable groups.

These concerns are substantiated by region-wide modeling efforts. A recent health impact assessment covering the broader MENA region, using bias-adjusted CMIP6 projections and Bayesian inference, estimated that under a high-emission scenario, the average heat-related mortality rate could reach approximately 123 deaths per 100,000 people per year by the end of the century [34]. Critically, the study found that the greatest relative increases in heat-related mortality are projected for the Arab states of the Persian Gulf—Qatar, Oman, and the UAE—despite their relative economic prosperity, while the largest absolute burdens are expected in Iran, Iraq, Palestine, and Saudi Arabia. These projections underscore the fact that no country within the EMME will be spared from escalating heat-related health risks, and that wealth alone does not confer immunity from climate-driven mortality.

Projections also indicate that climate change will alter the general characteristics of the climate in the region. For example, following the Köppen-Geiger classification system, Greece and Cyprus are expected to transition from semi-arid cold to semi-arid hot climates [35], and significant regional shifts in seasonal length are expected [36]. Climate model simulations suggest that summer periods, through expanding Persian Troughs (i.e., shallow thermal lows extending from the Indian monsoon region across Iran and the Arabian Peninsula, into the Eastern Mediterranean [37]), will continue to linger into traditionally cooler months [13]. In contrast, winter periods dominated by Cyprus Lows (i.e., mid-latitude lows that typically develop over the Eastern Mediterranean, transporting cool and moist air over the region from Eastern Europe over the warmer Mediterranean Seas [38]) are expected to become shorter. These changes in regional climate and weather patterns are likely to impact seasonal variability, ecosystems, biodiversity, and human well-being across the region [39].

### 2.2. Health impacts

#### 2.2.1. Weather patterns and seasonal health risks

Recent research efforts have focused on the epidemiological consequences of seasonal weather patterns [18],[40]–[42]. In this context, Assaf Hochman discussed whether different weather regimes (i.e., classifications of large-scale atmospheric circulation patterns defined by variables such as sea level pressure) could help predict variations in influenza cases during the winter. Results indicate a strong correlation between Cyprus Lows and peaks in confirmed flu cases and influenza-like illness (ILI) in the EMME region. A delay of 1–2 weeks between Cyprus Lows and flu peaks aligns with the appearance of typical symptoms and infection. A more advanced approach to capture the influence of synoptic weather patterns on heat risk uses the Climate Stress Index (CSI) during periods influenced by the Persian Trough [43]. Future projections estimate that more frequent and stronger heatwaves in the area are expected to raise heat-related mortality tenfold in Israel alone, particularly among elderly populations. Mortality rates exhibit a steep rise when CSI values exceed the 90th percentile, emphasizing the urgency of targeted adaptation strategies [43]. This suggests that using weather patterns in epidemiological models, instead of relying only on traditional meteorological variables, could improve the prediction of seasonal disease outbreaks, as well as heat-related morbidity and mortality [38].

#### 2.2.2. Accounting for physiological heat stress

To capture the full complexity of heat stress and its impact on human health, it is necessary to move beyond standard temperature-based measures. The modified Physiologically Equivalent Temperature (mPET) index is a more sophisticated indicator that accounts for both the environmental (e.g., radiant temperature, humidity, wind speed) and thermo-physiological (e.g., sex, age, weight, metabolic activity) factors that affect the human thermal perception [44],[45]. Christos Giannaros introduced a population-weighted mPET dataset, created for 72 regional units of Greece, that captures daily exposure and short-term acclimatization effects [44]. Unlike conventional datasets, which overlook population-wide variability (e.g., ERA5-HEAT), mPET provides a more precise representation of thermal exposure. To address the limited statistical power caused by small sample sizes, a hierarchical generalized additive model (GAM) was employed [46]. Results showed that heat-related mortality risk is strongly linked to prolonged exposure, with outdoor workers experiencing lethal heat stress even on non-extreme heat days. Importantly, these data reveal that some regions in Greece have recorded more than 60 days per warm season with dangerous levels of physiological heat stress [47].

An important application of mPET is in the framework of the HEAT-ALARM project [48], which issues heat warnings across Greece based on three criteria: (1) hot nights (exceeding mean temperature thresholds between 10 pm and 6 am), (2) extreme daytime heat stress linked to excess mortality, and (3) prolonged exposure (>6 h of strong heat stress). Using these criteria, specific heat stress warnings (strong/extreme/most extreme) are issued for each regional unit and population group. By integrating location and population-specific risks, this approach improves heat warning systems and adaptation strategies.

#### 2.2.3. Nighttime heat and health

While daytime heat is well studied, exposure to hot nights remains an often overlooked yet critical public health concern, especially considering the trend of increasing minimum temperatures. High nocturnal temperatures prevent the body from recovering, leading to sleep deprivation and heightened health risks, particularly among vulnerable groups [49].

Research on the impacts of nighttime heat is often based on minimum temperature (Tmin), which is typically recorded at sunrise. However, this approach does not capture the most stressful hours of the night, when the body is most sensitive to heat. To address this limitation, Roye (2017) introduced the Hot Night Duration (HNd) and Hot Night Excess (HNe) indices, which quantify both the percentage of nighttime hours exceeding a heat threshold and the total accumulated heat stress throughout the night [50]. These indices improve the assessment of prolonged night-time heat exposure by explaining the physiological consequences of sustained nocturnal thermal stress. Results show a strong link between hot nights and increased mortality, with HNe consistently showing higher risk estimates than HNd in Mediterranean cities [49],[50]. A study developed in Madrid during the 2017 heatwave focused on the effects of UHIs on nighttime temperatures. Results showed that a) nighttime heat excess indices increased rapidly with urban density and b) nighttime heat stress was lowest in open spaces, forests, and low-density areas with plenty of greenery. However, the same study suggested that while the UHI effect leads to hotter nights in urban centers, it is not the only factor, as broader regional climate patterns and land uses also contribute to nighttime thermal stress [51].

#### 2.2.4. Are we adapting?

Research shows that although temperatures keep rising in the EMME, its populations are not adapting to temperature extremes as effectively as populations in other regions, leaving them vulnerable to climate-related health risks. A health analysis in Greece and Cyprus revealed that the minimum mortality temperature (MMT) (the threshold above or below which mortality increases) has been decreasing over time. In Cyprus, MMT decreased from 26.3 °C (2004–2012) to 23.9 °C (2011–2019) and, in Athens, from 26 °C (1999–2007) to 21.8 °C (2011–2019). Anastasia Paschalidou, during her presentation, noted that such findings contradict adaptation signs, indicating higher sensitivity to heat. This pattern suggests maladaptation, likely driven by rapid warming that outpaces people's ability to adapt (both physiological and behavioral) and by other structural and socio-economic factors, such as poor housing and energy poverty.

Maladaptation, however, is not uniformly distributed across populations within a given country, and structural inequalities can produce sharply divergent vulnerability profiles even within the same urban environment. This is particularly evident in the Arab states of the Persian Gulf, where non-national migrant workers often constitute a substantial portion of the resident population and make up the majority of the outdoor workforce. Research from Kuwait revealed that these migrant workers face an elevated burden of heat-related mortality: In a time-series analysis, Alahmad and collaborators found that under extreme heat conditions, non-Kuwaitis of working-age (i.e., 15–64 years old) experienced a substantially higher relative risk of death than Kuwaiti nationals within the same age bracket (relative risk of death: 2.59 [95% CI: 1.20–5.59] vs 1.76 [95% CI: 0.63–4.91], respectively) [52].

The mass mortality observed during the 2024 Hajj pilgrimage in Mecca (Saudi Arabia) constitutes another example. During this event, temperatures reached over 51 °C at Mecca's Grand Mosque, resulting in at least 1300 fatalities and 2700 injuries. Among the deceased, approximately 83% were “pilgrims who had embarked on the journey without the necessary permits. That left them with little protection from the heat (e.g., access to air-conditioned facilities or food and water stations)” [53].

These examples highlight that adaptation, when assessed at the population-average level, can mask profound demographic inequities, and that effective heat-health policies in EMME countries must explicitly address the institutional and legal frameworks that shape heat exposure not only for the region's citizens but also for its temporary residents, manual laborers, and visitors.

Itamar Grotto and Hila Oren emphasized the need to improve how cities are designed, considering that urban design plays a critical role in influencing mortality and mental health risks. Recent findings from the ABCD study further suggest that extreme heat exposure may adversely affect mental well-being and cognitive functioning in children and adolescents, with disproportionate impacts observed among socioeconomically disadvantaged and minority populations [19],[54]. Given that technological adaptation, such as air conditioning, appears to have reached saturation, Grotto and Oren further highlighted the importance of inclusive, green public spaces in reducing health disparities, providing heat mitigation, and supporting well-being. Studies from New York and Tel Aviv show that access to greenery can improve mental health and birth outcomes, especially in lower-income communities [55],[56].

Although evidence suggests that green infrastructure can provide cooling and health benefits even in hot and arid urban environments [57],[58], the transferability of such findings across the EMME is not straightforward. Much of the evidence for nature-based urban cooling and health interventions originates from temperate climates with year-round precipitation; its direct application to the hot and dry conditions that characterize large parts of the EMME (including the Arabian Peninsula, inland Iran, and much of the Levant) requires careful adaptation to avoid pitfalls and unintended consequences [59]. For example, vegetation coverage that is excessively dense or poorly planned can trap longwave radiation or obstruct airflow, resulting in reduced comfort indicators [60]. Along the same lines, irrigation demands of urban greening in water-scarce settings should be calibrated to avoid competing with potable water requirements. These and other important caveats for regional adaptation are further addressed in Section 5.4.

### 2.3. Challenges in heat-health research

Despite clear evidence that climate change affects human health, significant challenges remain in assessing and addressing heat-related risks. Assaf Hochman noted that even though global projections often average extreme conditions among different regions (making them less useful at the local scale), regional and cross-border cooperation remains limited among EMME nations. Robin Fears highlighted the major data gaps and the limited heat-attributable mortality studies across the Middle East, Africa, and Asia [61]. In fact, a recent scoping review identified only 12 heat-health studies across the entire Arabian Peninsula published between 2010 and 2024, with 8 of them conducted in Kuwait, while the rest were spread across Saudi Arabia (4 studies), Qatar, Oman, Yemen, and the UAE (1 study each) [62]. This highlights an acute scarcity of heat-health data in key sections of the EMME where heat exposure is projected to be particularly intense, and where unique demographic structures (such as large migrant worker populations with high occupational heat exposure) require context-specific research. Similarly, Christos Giannaros emphasized the lack of long-term observational records for key environmental drivers like radiation, as well as the limitations of airport-based weather stations and gridded climate data, in capturing population exposure [44],[63].

Another important research challenge is evaluating the interaction between adaptation and mitigation strategies, to ensure that adaptation does not lead to maladaptation [14]. For example, Anastasia Paschalidou noted that increasing the use of air conditioning as an adaptation measure can raise air pollution, which can exacerbate health risks depending on how electricity is produced [64].

## 3. Vector-borne diseases

The complex interactions and compounding effects of climatic hazards, as well as non-climatic factors, are expected to have a major impact on the burden of VBDs in the coming years. The geographic distribution of invasive vector species is in a continuous state of flux, as they colonize new spaces, while at the same time, it has become increasingly difficult to predict the pathways through which these organisms, and the pathogens they transmit, will spread. Of particular importance for the EMME is the fact that all countries within the region present suitable environments for the establishment of invasive *Aedes* mosquito populations [14].

The workshop's session on VBDs featured presentations by Felipe Colon (Wellcome Trust, UK), George K. Christophides (Imperial College London, UK and The Cyprus Institute, Cyprus), Schlomit Paz (University of Haifa, Israel), John Vontas (Institute of Molecular Biology and Biotechnology/Foundation for Research and Technology Hellas, Greece), and Youmna M'ghirbi (Institut Pasteur de Tunis, Tunisia). These interventions described the impact of mosquito-borne disease outbreaks in regions previously void of their presence, as well as the re-emergence of previously eradicated diseases. The presenters expressed the necessity for a coordinated interdisciplinary effort with an urgent need for novel intervention strategies, the introduction of EWS and decision support systems (DSS), and regulatory roadmaps to enable access to technologies for rapid deployment.

### 3.1. Attribution versus association

One critical knowledge area addressed during the workshop was the lack of data available directly linking climate change to certain health outcomes. Although there is sufficient data to support the existence of associations between the incidence of infectious diseases and environmental/climatic factors, the evidence for direct attributions remains limited. In this regard, several speakers highlighted the complexities surrounding direct attribution, emphasizing the challenges in isolating climate change as the primary cause of specific health effects.

Felipe Colon pointed out that one of the major challenges for health impact attribution arose from the fact that, from over 3600 papers reviewed spanning the period from 2000 to 2024, only 10 had directly provided clear evidence of attributable climate impacts on health outcomes. Moreover, Schlomit Paz discussed the various factors that have been linked to increased mosquito activity and disease spread. While climate change, urbanization, and other factors have been associated with the expansion of mosquito-borne diseases, she emphasized the need to systemically collect epidemiological data on morbidity and mortality, along with climatic, environmental, ecological, and demographic data, to derive credible conclusions and accurately differentiate correlation and causation. Both researchers stressed the need to collect data from extensive epidemiological studies in order to find direct relations between specific climatic factors and their impact on health outcomes.

Following the presentations, the challenge of moving from association to attribution was heavily discussed. Some participants acknowledged that without clear attribution-level evidence, convincing decision-makers to justify specific interventions and accordingly allocate resources is difficult. To this end, emerging analytical approaches, including the integration of multiple climatic and non-climatic drivers through advanced modeling techniques, was proposed as a potential pathway toward more robust causal inference. Importantly, this was not a point of disagreement among speakers, but rather a shared recognition of a critical methodological gap that the field has yet to resolve. The need for improved data collection, broader geographic representation in the literature, with affected EMME countries currently underrepresented, and stronger collaboration between researchers and public health authorities were collectively identified as essential steps toward bridging the gap between association and attribution.

### 3.2. Early warning systems (EWS) and decision support systems (DSS)

During the 19th and 20th centuries, *Ae. aegypti* mosquito populations were well-established around the Mediterranean region and were identified as the primary vector involved in the deadly European outbreak of dengue virus between 1927–1928 [65],[66]. However, a combination of insecticide control, elimination of breeding sites, and improved sanitation resulted in the disappearance of this vector species from the region. Nevertheless, factors such as changing climatic conditions, urbanization, and the cross-boundary mobility of people and goods have enabled the reintroduction and establishment of *Ae. aegypti* populations in the EMME region [26],[67]. Furthermore, the widespread presence of *Ae. albopictus* (another important vector of human disease) in countries like Cyprus, Greece, Iran, Jordan, Lebanon, Israel, Palestine, and Syria [27],[68] indicates the essential need for the implementation of effective national and regional strategies for monitoring and surveillance [26],[69]–[71]. Implementing EWS will help track disease patterns, identify hotspots, and enable timely interventions to prevent widespread transmission of VBDs. During his intervention, John Vontas described how EWS can be guided by molecular diagnostics and systemic monitoring and discussed how the challenges associated with insecticide resistance include the limited number of efficient tools available for vector control, as well as the ability of vectors to adapt to these interventions. Therefore, there is a crucial need for enhancing the implementation of molecular and entomological evidence-based interventions, while at the same time, phasing out the indiscriminate (and often counterproductive) use of insecticides. Using proactive approaches such as entomological surveillance, resistance testing, and the application of precise, population-specific interventions is key to generating real-time data that can be used in developing EWS and predictive models for VDBs.

Given the complexity and uncertainty surrounding VBD outbreaks, driven by both climatic and non-climatic factors, DSS may offer a more actionable and accountable framework than conventional EWS. While the latter can alert to potential disease outbreaks based on entomological and environmental data, such alerts may lack actionable specificity and risk causing unnecessary public anxiety, particularly in the absence of clear causal links or established intervention pathways. In contrast, a DSS integrates epidemiological, entomological, climatic, ecological, and demographic data to inform evidence-based decision-making by governments and public health authorities. It supports proactive planning and targeted vector control, shifting the responsibility for action from tool developers or data generators to the appropriate regulatory and operational bodies. This distinction is critical: An EWS can unintentionally place the burden of response on both developers and exposed communities, especially when predicted risks do not materialize, potentially eroding public trust. A DSS, by design, empowers authorities with tailored guidance and accountability mechanisms to implement timely, effective measures, thus reducing the burden of VBDs through prevention rather than reaction. In practice, both systems can be integrated into an overarching scheme where information generated by an EWS is automatically captured and processed by the corresponding DSS, thereby improving the timeliness and accuracy of its output (Table 1).

**Table 1.** Comparison of early warning system (EWS) and decision support system (DSS).

	Early warning system (EWS)	Decision support system (DSS)
Primary function	Reduce transmission risk through timely detection, forecasting, and communication of potential disease outbreaks.	Reduce disease burden through prevention-oriented planning, targeted intervention, and evidence-based decision-making.
Focus	Hazard monitoring, forecasting, and communication of warnings.	Data analysis, scenario planning, prioritization of interventions, and support for decision-making across sectors.
Data inputs	Typically relies on entomological and environmental indicators, with the scope varying according to system capacity.	Integrates entomological, epidemiological, environmental, ecological, demographic, and operational data.
Guidance provided	Provides alerts or risk signals that can prompt preparedness and early action; intervention choices may require additional protocols, tools, or institutional processes.	Provides more tailored guidance, including intervention options, prioritization, and clearer operational pathways.
Role in preparedness capacity	Can serve as an important first step for settings with limited warning or decision-support infrastructure.	Represents a more comprehensive system that integrates warning functions with planning and response support.
Communication and public response	Can improve awareness and preparedness when alerts are timely, credible, and paired with clear communication.	Can support more targeted and contextualized communication by linking risk information to response options and planning.
Burden of response	Responsibility for interpretation and follow-up actions usually falls on public health agencies, local actors, or communities.	More explicitly distributes responsibility through predefined decision pathways and institutional response planning.
Public trust implications	Can build trust when warnings are accurate, actionable, and transparently communicated; trust may weaken if alerts are frequent, unclear, or not followed by visible action.	Designed to reinforce trust through structured accountability, transparent reasoning, and clearer links between evidence and action.

### 3.3. Intervention strategies

Whilst the use of insecticides and bed nets has been effective in reducing the risk of mosquito-borne disease transmission in some parts of the world, the efficacy of these methods is threatened by the growing spread of insecticide resistance, as well as the development of specific behavioral adaptations in mosquitoes [72]. In this context, the presentation by Youmna M'ghirbi described the successful eradication of malaria in Tunisia through a coordinated program, run between 1968 and 1979, which used diverse control strategies in combination with systemic monitoring to prevent re-establishment of autochthonous transmission. Unfortunately, the progressive anthropogenic degradation of the environment, changes in climatic factors, and disturbances to the local mosquito fauna, which are currently taking place in the region, enhance the threat of malaria re-emergence. Therefore, it becomes imperative to develop new tools and strategies to complement current control efforts.

During his talk, George Christophides addressed the use of genetically modified insects as innovative control tools that offer a promising avenue by altering the vector's ability to transmit diseases. One example of this is the recent generation of a transgenic strain of *Anopheles gambiae* (one of the most important vectors of malaria in the African continent), which was engineered to bias the inheritance of two exogenous antimicrobial peptides, whereby following ingestion of a blood meal, they would display antiparasitic effects by significantly delaying the parasite's development inside the vector [73]. As is the case with all genetic control methods, open-field implementation of this technology would require extensive risk assessments.

### 3.4. Regulatory roadmaps and guidelines

Technical guidelines and a roadmap are necessary to ensure effective implementation of disease control measures and limit the spread of VBDs. From the open discussions that followed the presentations, a clear point of tension that emerged was the gap between the availability of tools and the capacity to implement them. Using Cyprus as a case study, participants noted that despite the recent introduction and establishment of both *Ae. aegypti* and *Ae. albopictus*, the country currently lacks a national roadmap for responding to invasive vector species, including clear guidance on what interventions to deploy, when, and where, with well-intentioned actions risking being misdirected or even counterproductive. As one participant noted, applying the wrong intervention in the wrong context can produce adverse outcomes and may exacerbate the problem rather than contain it.

In this context, the introduction of standardized guidelines like the ones utilized by the pharmaceutical or agricultural industries was therefore proposed, outlining specific recommendations for intervention selection and deployment. These guidelines should outline what, when, where, and which interventions should be applied, and must be specific to the locality in which they will be introduced. Regulatory roadblocks, particularly around novel tools such as GM insects and irradiation-based approaches, were highlighted as significant barriers to implementation. The question of whether full regulatory harmonization across EMME countries is needed, or whether a more practical mapping of existing national frameworks would be sufficient, was briefly raised but not resolved. Participants agreed that since vectors do not respect national boundaries, regional coordination is essential. To this effect, it is crucial to liaise and coordinate efforts with relevant stakeholders (e.g., public health authorities and other government instances, researchers, and local community representatives), thereby improving data acquisition, curation, and sharing.

## 4. Air pollution

Climate change and air pollution are emerging environmental risk factors in the EMME region. This section discusses recent advancements in understanding the health effects of air pollution and the uncertainties associated with estimating excess mortality rates. It addresses the role of atmospheric modeling, the combined effects of heat and air pollution, and the role of particles' differential toxicity. Furthermore, it introduces the emerging concept of the environmental exposome, as well as effective strategies for managing human exposure after dust events. Presentations were offered by Jonilda Kushta (The Cyprus Institute), Jos Lelieveld (The Cyprus Institute, Max Planck Institute for Chemistry), Omar Hadad (University Medical Center Mainz), Nikolaos Mihalopoulos (National Observatory of Athens), Andrea Pozzer (Max Planck Institute for Chemistry and The Cyprus Institute), Panayiotis Kouis (University of Cyprus), and Wiessam Abu Ahmad (Hebrew University of Jerusalem).

### 4.1. Modeling uncertainties

During her presentation, Jonilda Kushta addressed the uncertainties associated with estimating excess mortality attributable to environmental exposure to fine and coarse particulate matter (PM_2.5_ and PM_10_, respectively). Methodologies utilized to estimate PM_2.5_ exposure include ground-based observations, satellite data, air quality models, and hybrid approaches. Atmospheric modeling tools prove advantageous for bridging data gaps in areas that lack station-based observations and for examining the consequences of differing pollution scenarios.

While air quality models are subject to limitations, such as coarse horizontal resolution [74] and the quality of input data (including anthropogenic emissions) [75], the uncertainties stemming from the model's horizontal resolution contribute marginally (approximately 8%) to the overall uncertainty in excess mortality predictions. In contrast, uncertainties related to relative risks (RRs) account for a significant proportion (approximately 30%) of the total uncertainty. In addition, the toxicity of PM_2.5_ components is not typically accounted for in the RRs, despite the growing evidence that anthropogenic PM_2.5_ (e.g., carbonaceous aerosols from road transportation and combustion sources) is more toxic than PM_2.5_ emitted from natural sources [76]. Furthermore, during health impact assessments, it is important to account for the interaction between air pollution and temperature effects, especially in urban areas, where urban heat island (UHI) and urban pollution island (UPI) effects coincide [77].

The interaction between UHI and UPI (e.g., through enhanced photochemical processes due to increased heat levels leading to elevated pollutants) becomes particularly important during heat waves. Subsequently, the synergistic effects of particulate matter and air temperature on human health contribute to the associated mortality. These factors introduce additional complexity in the assessment of the health effects. High thermal stress levels (maximum apparent temperature above 33 °C) favor pollution build-up [77]. In addition, pollutants like PM_10_, NO_2_, and O_3_ increased heat-related deaths in Thessaloniki by 47.7%, 38.7%, and 32%, respectively. All of the above underscore the need for a comprehensive approach to assess health burden, which includes the interaction of additional factors such as the PM_2.5_'s differential toxicity and thermal effects.

### 4.2. Excess mortality

Exposure to ambient air pollution causes both acute and chronic health effects [78]. Jos Lelieveld's presentation focused on the health effects and global excess mortality associated with air pollution from fossil fuel consumption. Acute effects include respiratory symptoms, coughing, eye irritation, and irregular heartbeat [79]. Prolonged exposure to PM_2.5_ can induce systemic inflammation and oxidative stress, ultimately leading to a range of health impairments, including ischemic heart disease, chronic obstructive pulmonary disease (COPD), asthma, lung cancer, and type 2 diabetes [79].

Approximately 8.3 million (95% CI: 5.6–11.2) excess deaths annually are attributable to PM_2.5_ and ozone (O_3_) pollution worldwide, with 5.1 million (95% CI: 3.6–6.3) deaths specifically linked to fossil fuel usage [78]. During his intervention, Jos Lelieveld highlighted that up to 80% of anthropogenic (i.e., avoidable) fatalities could be prevented through the elimination of fossil fuel emissions, pointing out that regions such as Eastern Europe and Italy exhibit elevated mortality rates due to air pollution. Furthermore, countries with additional pollution sources, such as the domestic burning of solid biofuels and natural emissions in Sub-Saharan Africa, North Africa, and the Middle East, continue to experience significant excess mortality and benefit less from reductions in fossil fuel emissions [78].

Cardiovascular diseases account for over 50% of global excess mortality attributable to air pollution, followed by pulmonary diseases [80]. Child mortality, particularly due to pneumonia, is a pressing issue in low-income countries and significantly affects life expectancy estimates. Interestingly, recent studies have revealed that the implementation of air pollution control measures can successfully reduce mortality: A recent publication by Georgulias and collaborators, focused on China, showed that within a span of two decades, the reduction of PM_2.5_ at the levels of 2018–2019 prevented over 1 million excess deaths per year, and avoided an average reduction in life expectancy of over one year [81].

### 4.3. The environmental exposome

First proposed by Christopher Wild in 2005, the term *exposome* is defined as the compilation of all “life-course environmental exposures (including lifestyle factors), from the prenatal period onwards” [82]. Environmental exposures lead to endogenous biochemical and functional changes and are often associated with adverse health outcomes [83],[84]. Exposures to environmental factors (e.g., built environment, climate, traffic, and air pollution) are a significant part of the general external exposome and exert an important influence on human morbidity and mortality—often considered to be more important than genetic predisposition [83]. We must clarify that although the exposome is a broad, integrative framework that goes well beyond air pollution, it is discussed in this section because it was introduced during the session on air quality as a means of contextualizing the cumulative health effects of multiple environmental stressors.

During his talk, Omar Hadad introduced the Gutenberg Health Study (GHS) (www.unimedizin-mainz.de/ghs/en/overview-1.html), a comprehensive cohort study aimed at investigating the health effects of air pollution and various environmental risk factors, including noise and heat. The GHS has tracked 15,000 individuals since 2007 to explore the development of chronic diseases, such as cardiovascular diseases, cancer, immune system disorders, metabolic diseases, and mental health conditions. The primary objective of the GHS is to elucidate the underlying mechanisms of disease, while identifying both risk and protective factors [84]. The dataset from the GHS was integrated with diverse environmental data to establish an environmental exposome atlas. He suggested that similar studies in the EMME region could significantly improve public health, since the EMME is one of the world's most polluted regions, with significant urban growth and environmental health concerns.

Several other studies have indicated an increased risk of cardiovascular disorders attributed to noise annoyance [85],[86]. Additionally, a growing number of studies have started to evaluate the combined effects of multiple environmental risk factors, such as air pollution, light pollution, traffic noise, and lack of green spaces, on non-communicable diseases, including cardiovascular diseases and type 2 diabetes [84],[87]–[89]. Since these environmental exposures are often co-localized, especially in urban areas, the studies highlight the need to evaluate the cumulative risk of a disease from exposure to multiple environmental exposures rather than to a single exposure [84]. However, adverse health effects are not limited to cardiovascular disorders. The interaction between high temperatures, PM_2.5_ pollution, and health effects has a critical role in the prevalence of adverse pregnancy outcomes like pre-term births [90].

The presentation by Wiessam Abu Ahmad showed that during hot periods, exposure to even low PM_2.5_ concentrations can result in an increased risk of pre-term births and miscarriages. High temperatures and sunlight intensity may be related to an alteration of the chemical composition of PM_2.5_ particles, potentially making them more reactive and hazardous to human health. The enhanced photochemical formation of secondary particulate matter, including sulfates, nitrates, and organic aerosols, often coincident with high-temperature conditions, increases the potential for adverse health effects (e.g., higher risk of adverse birth outcomes) [90]. This is specifically important for climate change mitigation policies, which should take into account the interaction between high temperature and PM_2.5_ pollution.

### 4.4. Particulate matter toxicity and health effects

The differential toxicity of PM_2.5_ plays a significant role in its effects on human health. The particles' composition and toxicity differ between sources, regions, and periods. However, current regulations do not account for these differences. Nikos Mihalopoulos highlighted that semi-volatile organic carbon (SVOC) compounds derived from fossil fuels and fire emissions exhibit significant toxicity. He addressed the differential toxicity of polycyclic aromatic hydrocarbons (PAHs) and oxygenated PAHs (OPAHs), both of which are recognized for their genotoxic, mutagenic, and carcinogenic properties.

The European Union currently establishes safety limits exclusively for Benzo(a)Pyrene (BaP), with an annual concentration limit set at 1 ng/m^3^. After two field campaigns conducted during the summer and winter seasons across six Greek cities (Ioannina, Athens, Piraeus, Volos, Xanthi, and Heraklion), it was revealed that all six exhibited elevated levels of PAHs, exceeding the established EU safety thresholds [91]–[93]. This underscores the imperative for comprehensive monitoring of additional toxic compounds beyond the current EU standards, to prevent significant underestimation of PAH toxicity and the associated carcinogenic risks, which are currently underestimated.

Oxidative potential (OP) serves as an important measure of the oxidative capacity of PM_2.5_, offering a more relevant metric for health exposure than PM_2.5_ mass. In the cities studied, OP was predominantly influenced by local sources, such as wood burning in winter, and by regional pollution and traffic (particularly from non-exhaust emissions) in summer [92]. These findings highlight the necessity of reducing such emissions to effectively enhance air quality in the eastern Mediterranean region and suggest that a focus on PM_2.5_ mass concentration alone is inadequate for effectively assessing exposure risks and mitigating the differential toxicity of these pollutants.

Evidence shows that the harmfulness of particles is highly dependent on their size [94],[95]. Ultrafine particles (UFPs) are defined as those with an aerodynamic diameter smaller than 0.1 µm. Due to their capacity to penetrate the bloodstream and spread throughout the human body, UFPs are deemed particularly hazardous. Machine learning models have been found to be effective tools in estimating UFP concentrations and their health effects. For example, Georgiades and colleagues constructed the first global, high-resolution (1 km^2^) estimates of particle number concentrations (PNC) and UFP through machine learning tools, which can be used to assess the relationship between UFP exposure and excess mortality [96]. The dataset captures fine-scale spatial heterogeneity, such as urban hotspots, which is highly important for the increasing urbanization in the EMME region.

Dust storms represent a significant source of PM_2.5_ and PM_10_ in the Mediterranean region and are anticipated to become more frequent, potentially as a result of climate change. The presentation by Panagiotis Kouis highlighted the health risks associated with exposure to dust storms, which include cardiovascular and respiratory diseases, thereby underscoring the necessity for practical and sustainable guidelines for exposure reduction. His research team conducted an evaluation of specific guidelines in Cyprus and Greece, utilizing randomized intervention trials involving asthmatic children and adults with paroxysmal atrial fibrillation. The guidelines supported the limitation of outdoor exposure, reduction of physical activity, and implementation of measures to enhance indoor air quality, such as the use of air purifiers to mitigate pollutant infiltration. The findings indicate that the application of these exposure reduction guidelines can significantly reduce exposure to dust and air pollutants, while improving health outcomes during events of elevated airborne dust [97],[98].

## 5. Policy implications and suggestions

As evidenced in the previous sections, extreme temperatures, air pollution, and VBDs constitute distinct yet related threats that are exacerbated by climate change. The interconnected and transboundary nature of these challenges demands a concerted and comprehensive approach across the region in order to address them.

From the information provided during the workshop, it is possible to identify several crucial principles for building climate resilience and protecting public health in urban areas across the EMME. These are briefly described below and summarized in Figure 3.

### 5.1. Fostering collaboration and open communication

Effective climate action requires dynamic, cross-border cooperation among countries sharing common climate risks. In the EMME, where countries often lack normalized diplomatic relations or face active conflicts and closed borders, yet share airsheds, water resources, and the risks posed by extreme climate events, this cooperation cannot rely solely on conventional diplomatic or academic channels. It is therefore necessary to develop flexible cooperation mechanisms (such as technical working groups hosted by multilateral bodies, shared digital platforms for surveillance data, and joint research consortia operating under the auspices of international organizations) that can function even when direct bilateral relations are constrained. Additionally, involving policymakers, public health practitioners, economists, local communities, and scientists, from the inception of research projects and policy design, ensures that interventions are not only context-specific and widely accepted but also that their expected outputs are aligned with local and national priorities.

**Figure 3.**
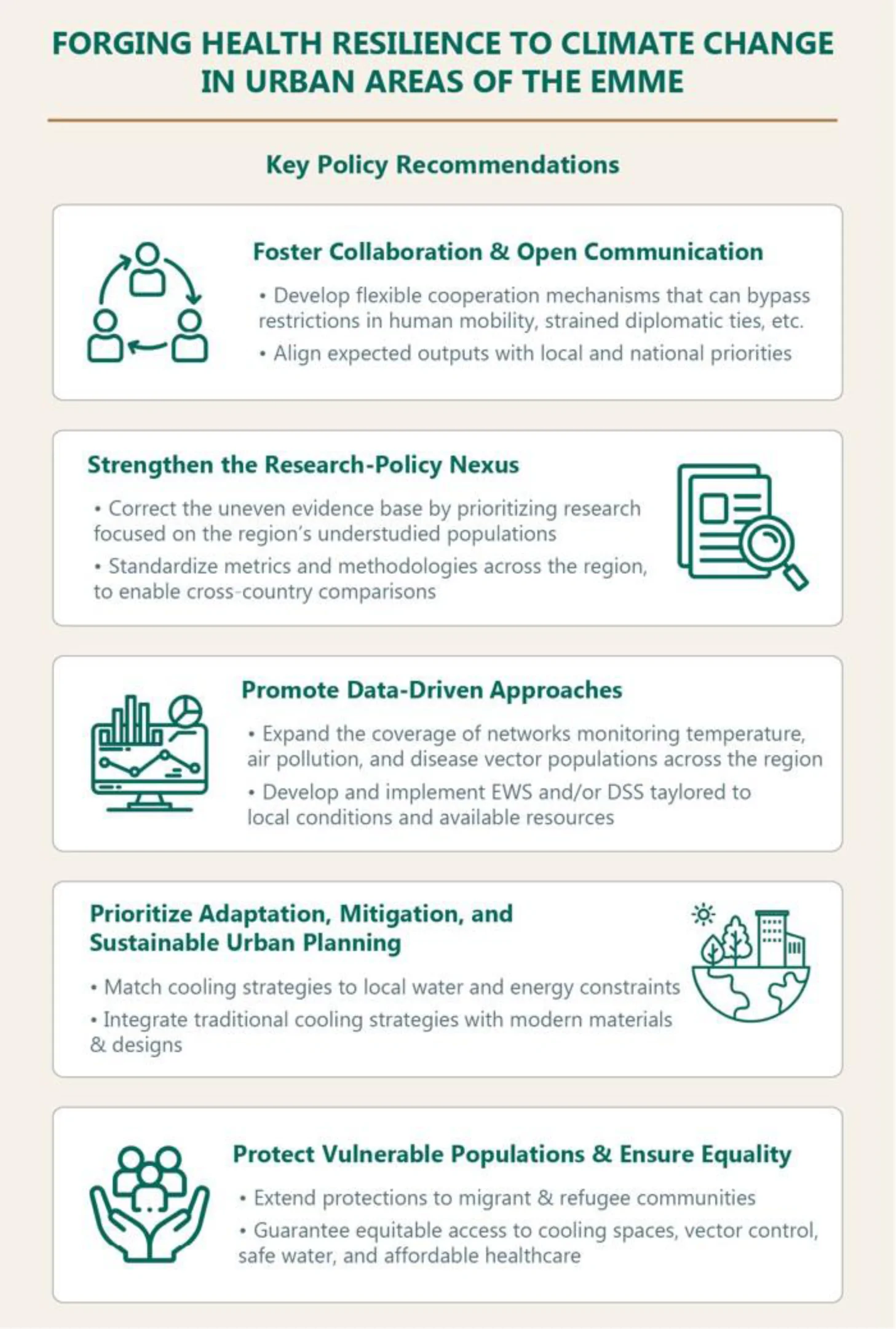
Visual summary of key policy recommendations. These suggestions aim at fostering coordinated, effective, and equitable responses to the health challenges posed by climate change in urban areas of the EMME. Figure created using open-access graphic elements from flaticon.com.

### 5.2. Strengthening the research–policy nexus and evidence-based action

A recurrent theme across the workshop's presentations was the critical need to bridge the gap between scientific research and policy implementation. Several participants underscored the need to move beyond mere problem acknowledgment and into proactively drafting comprehensive and effective strategies, informed by teams of experts in relevant fields from both the natural and social sciences, as well as other stakeholders. However, to achieve this, it is imperative to address the marked unevenness of the existing evidence base within the EMME. As discussed in Section 2.3, heat-health attribution studies remain scarce for several countries in this region, with similar gaps in the literature covering air pollution and vector-borne diseases. Bridging the research-policy gap, therefore, requires not only better integration of available evidence but also a deliberate prioritization of research focused on the region's understudied populations and settings, particularly where projected exposures are most severe and where context-specific data are most urgently needed to inform local policy.

Furthermore, in order to address potential inconsistencies in impact assessments across the region, it becomes necessary to standardize metrics and methodologies, improve data integration, and enhance model calibration, thereby ensuring accuracy and comparability across studies.

### 5.3. Promoting the use of data-driven approaches for improving monitoring strategies, EWS, and DDS

Timely access to accurate information is a fundamental component of any preparedness and response strategy. In this context, regional climate resilience in the EMME could greatly benefit from expanding the coverage of systems for monitoring key factors such as temperature, air pollutants (including specific toxins, like PAHs), and vector populations. Integrating climate, environmental, and health data to develop and implement effective EWS can help identify emerging risks, track disease patterns, and enable timely interventions across multiple environmental threats. However, while EWS are useful for signaling potential hazards, DSS are especially critical for complex challenges such as VBDs. By combining local climate, environmental, entomological, and health data, DSS enable authorities to interpret signals, assess response options, and implement targeted interventions while optimizing available resources. Given the highly nonlinear and context-dependent nature of VBD transmission, DSS offer an actionable and responsive framework for anticipating outbreaks and guiding policy decisions.

### 5.4. Prioritizing adaptation, mitigation, and sustainable urban planning

Long-term resilience requires a dual focus on adapting to current changes while, at the same time, mitigating future risks. Therefore, when implementing adaptation measures (e.g., energy-efficient housing, green infrastructure, indoor air purification), it is of paramount importance to carefully evaluate potential trade-offs, such as increased energy consumption or unintended ecological impacts, in order to avoid the risk of maladaptation.

Additionally, it is crucial to recognize the profound interconnections that exist between urban design and public health. To this effect, strategies such as the promotion of green infrastructure, carefully designed inclusive public spaces, and energy-efficient buildings can greatly contribute to mitigating challenges that affect urban areas in particular, such as heat island effects, exposure to air pollutants, and the proliferation of breeding grounds for certain disease vectors. However, the design of these interventions must be calibrated to local climatic, hydrological, and cultural conditions. As Negev and colleagues argued in their analysis of city design in hot and dry climates [59], nature-based and water-sensitive urban design strategies developed for temperate, year-round rainfall settings frequently translate poorly to the EMME, where precipitation is concentrated in a short winter season, followed by several months with no rain. In Gulf and inland desert cities, irrigation-dependent greening can compete with already-strained municipal water supplies and may yield only marginal cooling benefits relative to its resource cost.

A context-appropriate adaptation strategy for arid parts of the EMME should therefore complement conventional green infrastructure with a broader portfolio of interventions. First, drought-resistant native vegetation arranged to optimize shading rather than canopy density can deliver meaningful pedestrian-level cooling. Research from Egypt, Saudi Arabia, and Algeria reports reductions in mean radiant temperature of 20–30 °C under dense tree shading [60]. Second, high-albedo pavements and reflective building surfaces, paired with appropriate shading geometry to avoid undesirable radiative effects, can reduce surface temperatures by up to 7 °C in hot-arid contexts [99]. Third, the revival and modernization of vernacular passive-cooling design elements with a long regional history, such as courtyards and windcatchers (also known as *Badgir* in Persian), which have repeatedly been shown to reduce indoor and pedestrian thermal stress with minimal energy demands [100],[101]. Beyond their thermal performance, such interventions also have the advantage of being culturally acceptable and historically rooted in many of the region's urban contexts, which can facilitate community adoption and long-term maintenance—considerations that are too often absent from purely technocratic adaptation strategies [59].

### 5.5. Protecting vulnerable populations and ensuring equity

It is well known that climate change disproportionately affects certain segments of the population, including the elderly, individuals with preexisting health conditions, outdoor workers, and those in socioeconomically disadvantaged groups, such as migrants and refugees, who are often the most exposed and least able to adapt. Therefore, policies aimed at improving climate resilience in the EMME should include provisions that specifically target these high-risk groups, ensuring equitable access to green spaces, cooling stations, vector control, safe water, and affordable healthcare.

## 6. Concluding remarks

Urban environments within the EMME confront an accelerated convergence of climate-driven health threats, demanding a nuanced understanding beyond generalized climate impact narratives. In this context, the workshop on *Climate Change and Health in the Urban Environment* provided an ideal framing for the exchange of novel information and cross-fertilization of ideas among researchers from various disciplines working on the EMME region.

Regarding the risks posed by extreme temperatures, the workshop's participants underscored the fact that often-underestimated factors, such as rising heat during nighttime, as well as the observed trend of decreasing minimum mortality temperatures, suggest potential maladaptation in regional populations, rather than effective acclimatization. The documented decline in MMT across EMME cities over extended periods of dangerous physiological heat stress experienced by outdoor workers across the region demonstrates that our ability to address these challenges is currently restricted by the limitations of predictive models, which often fail to adequately capture factors such as population-specific vulnerabilities and the nuances of thermal stress beyond simple temperature metrics. Participants also emphasized that adaptation measures may involve important trade-offs, particularly in water-scarce and rapidly urbanizing environments across the EMME region. Taken together, these findings underscore the need to move beyond generic heat action plans toward population- and location-specific early-warning systems that account for the heterogeneous vulnerability profiles of EMME populations.

The workshop also highlighted the shifting landscape of VBDs, with climate change facilitating the expansion of vectors into new territories within the EMME. A critical scientific challenge lies in moving from mere observed associations toward the direct attribution of the influence that climatic factors exert on VBD epidemiology. In this regard, the recent establishment of invasive *Ae. aegypti* and *Ae. albopictus* populations across multiple EMME countries, in many cases in the absence of national roadmaps for responding to their presence, concretely illustrates the consequences of this gap. The spread of insecticide resistance further complicates control efforts, pointing toward the need for sophisticated EWS and DSS, as well as other innovative interventions, potentially including genetic strategies guided by clear regulatory frameworks, possibly similar to those used in the pharmaceutical and agricultural industries.

Interestingly, the workshop's first tangible collaborative output was produced in the context of invasive disease vectors. During the discussion session devoted to VBDs, we identified a crucial knowledge gap regarding the *Ae. aegypti* population that had recently invaded Cyprus: While traditional vector control strategies rely heavily on the use of chemical insecticides, no data existed regarding the susceptibility of this mosquito population to pyrethroids, the only class of insecticide allowed for routine mosquito control under EU regulations [102]. To address this, workshop attendees Marco Neira (Cyprus) and John Vontas (Greece) designed a strategy that included coordination and collaboration with national health authorities in Cyprus, as well as the drafting and submission of a collaborative proposal to the InfraVec2 consortium (https://infravec.org/) in order to obtain the required funding. Following the approval of this proposal, various research groups joined efforts and performed a thorough genotypic and phenotypic screening of Cypriot *Ae. aegypti*, characterizing the levels of resistance observed and identifying the genetic mechanisms that are potentially associated with said resistance. A comprehensive report of the results was provided to the Cypriot Ministry of Health, and a research paper was submitted for publication (undergoing peer review at the time of the drafting of this manuscript). This experience illustrates how a focused scientific gathering can translate directly into coordinated, cross-border research that informs national public health policy. We hope it will serve as a model—and a catalyst—for further collaborative initiatives emerging from this and similar events, progressively strengthening the network of climate-health cooperation across the EMME.

Air quality deterioration was discussed as a third critical intersecting challenge. Although the health burden from particulate matter in the region is significant, important uncertainties need to be resolved to improve modeling efforts, especially concerning the differential toxicity of particle components based on source and size (e.g., anthropogenic PM_2.5_, ultrafine particles, and PAHs from wood burning) and the relative risks they pose. Evidence from across the region showing that PAH concentrations frequently exceed established safety thresholds, while current regulations address only a fraction of known toxic particle components, highlights that reliance on PM_2.5_ mass concentration alone is insufficient. In this context, it is recommended that future research takes into consideration the cumulative and synergistic effects that can result from multiple, co-existing environmental stressors (i.e., the exposome), including air pollution, heat, and noise, particularly in densely populated urban settings.

We wrap up by proposing a series of recommendations for informing policy decisions in the EMME. By adopting these overarching principles, policymakers can devise a more coordinated, effective, and equitable response to the complex health challenges posed by climate change. Placing emphasis across all areas on data-driven decisions, proactive collaborations, and a balance between immediate needs (adaptation) and long-term sustainability (mitigation) offers a robust framework for future action.

Finally, we acknowledge that the limited geographic coverage of the pool of workshop participants constitutes an important limitation of the event, which is therefore reflected in this synthesis. As stated in the introduction, we sought to mitigate this situation here by referencing relevant published data from regions that were underrepresented in the workshop; we recognize, however, that secondary engagement with the literature cannot fully substitute for direct, in-person participation. In Section 5.1, we call upon the regional community to pre-empt future challenges of this nature by implementing robust cross-boundary collaboration mechanisms, including partnerships with regional and/or international bodies that can provide logistic support and enhance professional networks.

## Use of AI tools declaration

The authors declare they have not used Artificial Intelligence (AI) tools in the creation of this article.
